# Neutrophil-specific knockout demonstrates a role for mitochondria in regulating neutrophil motility in zebrafish

**DOI:** 10.1242/dmm.033027

**Published:** 2018-03-01

**Authors:** Wenqing Zhou, Lingyan Cao, Jacob Jeffries, Xiaoguang Zhu, Christopher J. Staiger, Qing Deng

**Affiliations:** 1 Department of Biological Sciences, Purdue University, West Lafayette, IN 47907, USA; 2 Department of Botany and Plant Pathology, Purdue University, West Lafayette, IN 47907, USA; 3 Purdue Institute for Inflammation, Immunology, & Infectious Disease, Purdue University, West Lafayette, IN 47907, USA; 4 Purdue University Center for Cancer Research, Purdue University, West Lafayette, IN 47907, USA

**Keywords:** Neutrophil, Mitochondria, Cell migration, Tissue-specific knockout, Zebrafish

## Abstract

Neutrophils are fast-moving cells essential for host immune functions. Although they primarily rely on glycolysis for ATP, isolated primary human neutrophils depend on mitochondrial membrane potential for chemotaxis. However, it is not known whether mitochondria regulate neutrophil motility *in vivo*, and the underlying molecular mechanisms remain obscure. Here, we visualized mitochondria in an interconnected network that localizes to the front and rear of migrating neutrophils using a novel transgenic zebrafish line. To disrupt mitochondrial function genetically, we established a gateway system harboring the CRISPR/Cas9 elements for tissue-specific knockout. In a transgenic line, neutrophil-specific disruption of mitochondrial DNA polymerase, *polg*, significantly reduced the velocity of neutrophil interstitial migration. In addition, inhibiting the mitochondrial electron transport chain or the enzymes that reduce mitochondrial reactive oxygen species also inhibited neutrophil motility. The reduced cell motility that resulted from neutrophil-specific knockout of *sod1* was rescued with *sod1* mRNA overexpression, or by treating with scavengers of reactive oxygen species. Together, our work has provided the first *in vivo* evidence that mitochondria regulate neutrophil motility, as well as tools for the functional characterization of mitochondria-related genes in neutrophils and insights into immune deficiency seen in patients with primary mitochondrial disorders.

This article has an associated First Person interview with the first author of the paper.

## Abstract (precis)

**Summary:** Using CRISPR/Cas9-mediated neutrophil-specific knockout, a requirement for mitochondrial DNA polymerase, electron transportation chain and superoxide dismutases in neutrophil migration is demonstrated in zebrafish, providing genetic evidence of the immunodeficiency seen in mitochondrial disease patients.

## INTRODUCTION

Cell motility, crucial for development, immunity, wound healing and cancer metastasis, is central to homeostasis in both health and disease. Mitochondria are the powerhouse for energy production and integrated regulators for cell signaling, modulating intracellular calcium concentration and apoptosis, to produce metabolic intermediates and reactive oxygen species (ROS). Polarized localization of mitochondria is observed in isolated cancer cells and lymphocytes in culture and is thought to drive cell migration, presumably by localized ATP production (Bao et al., 2015; Campello et al., 2006; Desai et al., 2013). In addition, mitochondria regulate redox and calcium signals that are also relevant to cell migration.

Neutrophils are fast-moving cells and the first line of defense against invading pathogens. Polarized localization of the actin cytoskeleton and intracellular signaling molecules is essential for neutrophil motility, chemotaxis and immune functions. It has long been appreciated that neutrophils obtain the energy required for function through glycolysis in an oxygenated environment to produce ATP, in a process known as the Warburg effect (Maianski et al., 2004; Warburg, 1956). Isolated primary human neutrophils have a partially dysfunctional mitochondrial network, with low expression of electron transport chain components, and low rates of respiration and ATP production, yet are able to maintain mitochondrial membrane potential. Disrupting mitochondria membrane potential or prolonged inhibition of the F0-ATPase inhibited neutrophil chemotaxis in 2D, without inducing cell apoptosis or inhibiting the activation of the respiratory burst or phagocytosis (Bao et al., 2015; Fossati et al., 2003). Of note, disrupting the mitochondria membrane potential resulted in a much more profound phenotype than inhibiting F0-ATPase activity alone (Fossati et al., 2003), suggesting that mitochondria possibly also regulate neutrophil chemotaxis via mechanisms other than ATP production. In addition, previous studies were limited by the dependency in pharmacological inhibitors, owing to the fact that neutrophils are terminally differentiated and not genetically tractable. Genetic evidence for the importance of mitochondria-related pathways in neutrophil chemotaxis is scarce.

It is well accepted that leukocytes *in vitro* and in tissue display distinct behaviors and might utilize separate signaling mechanisms (Lämmermann et al., 2008; Yamahashi et al., 2015). The biological relevance of mitochondria for neutrophil migration *in vivo* has not been characterized. It is noted that patients with primary mitochondrial disorders are indeed highly susceptible to opportunistic bacterial and fungal infections that are typically controlled by neutrophils (Walker et al., 2014). The lack of an *in vivo* model significantly hinders the mechanistic characterization of how mitochondria regulate neutrophil function.

Zebrafish are a well-established model to study neutrophil biology (Deng and Huttenlocher, 2012). However, a lack of standard tissue-specific knockdown or knockout technique in the zebrafish field has limited the power of this model organism when studying developmental essential genes, such as those related to mitochondrial function. Recently, a tissue-specific knockout approach has been developed by the Zon group (Ablain et al., 2015). Here, we have further adapted this system for use in zebrafish neutrophils. We have successfully disrupted mitochondrial DNA polymerase in neutrophils, thereby providing the first evidence that the mitochondrial network plays an indispensable role in regulating neutrophil motility *in vivo*.

## RESULTS

### Mitochondrial dynamics in migrating neutrophils

**Fig. 1.**
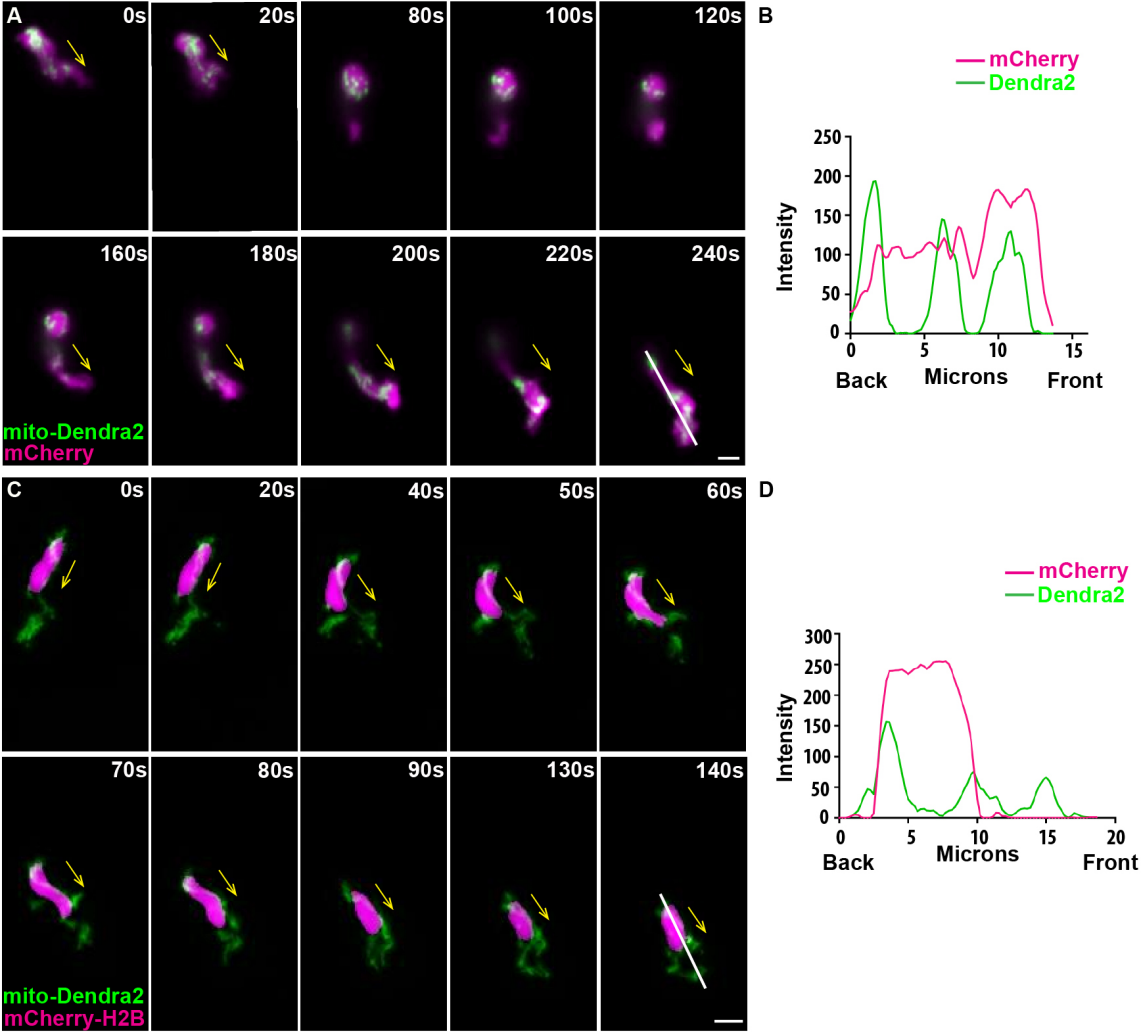
**Mitochondria localize to both the front and the rear of neutrophils.** (A) Random motility of neutrophils in the head mesenchyme of a 3 dpf larvae of *Tg(lyzC:mitoDendra2)^p14^* crossed with *Tg(lyzC:mCherry)*. (B) Quantification of fluorescence intensity along the indicated line (at 240 s). (C) *Tg(lyzC:mitoDendra2)^pu14^* was crossed with *Tg(lyzC:mCherry-H2B)* to visualize mitochondria network relative to the nucleus. (D) Quantification of fluorescence intensity along the indicated line (at 140 s). Montages of one representative movie out of eight are shown. Arrows indicate the direction of cell migration. Scale bars: 5 µm.

Mitochondria are morphologically dynamic organelles that travel along microtubules (Heggeness et al., 1978), and continuously divide and fuse to form small structures or interconnected networks. The localization of mitochondria in a migrating cell provides significant insight into the potential role of mitochondrial network. To observe mitochondrial dynamics in migrating zebrafish neutrophils, we generated a transgenic zebrafish line, *Tg(lyzC:mitoDendra2)^pu14^*, expressing a green fluorescent protein, Dendra2, fused to a mitochondria-localization sequence. Total neutrophil numbers, distribution and motility were comparable with the standard *Tg(lyzC:GFP)* control line (Hall et al., 2007). A spinning disk confocal was used to capture the morphology and localization of the mitochondrial network in the randomly migrating cells in the head mesenchyme of the 3 days postfertilization (dpf) larvae (Lam et al., 2014). Mitochondria primarily formed a tubular structure that localized to the front and rear relative to the nucleus, but did not necessarily enter cell protrusions or the uropods (Fig. 1; Movies 1 and 2). Similar observations were made when a neutrophil reversed the direction during random migration (Movie 3) or during the neutrophil wound response (Movie 4).

### A gateway cloning system for tissue-specific knockout

**Fig. 2.**
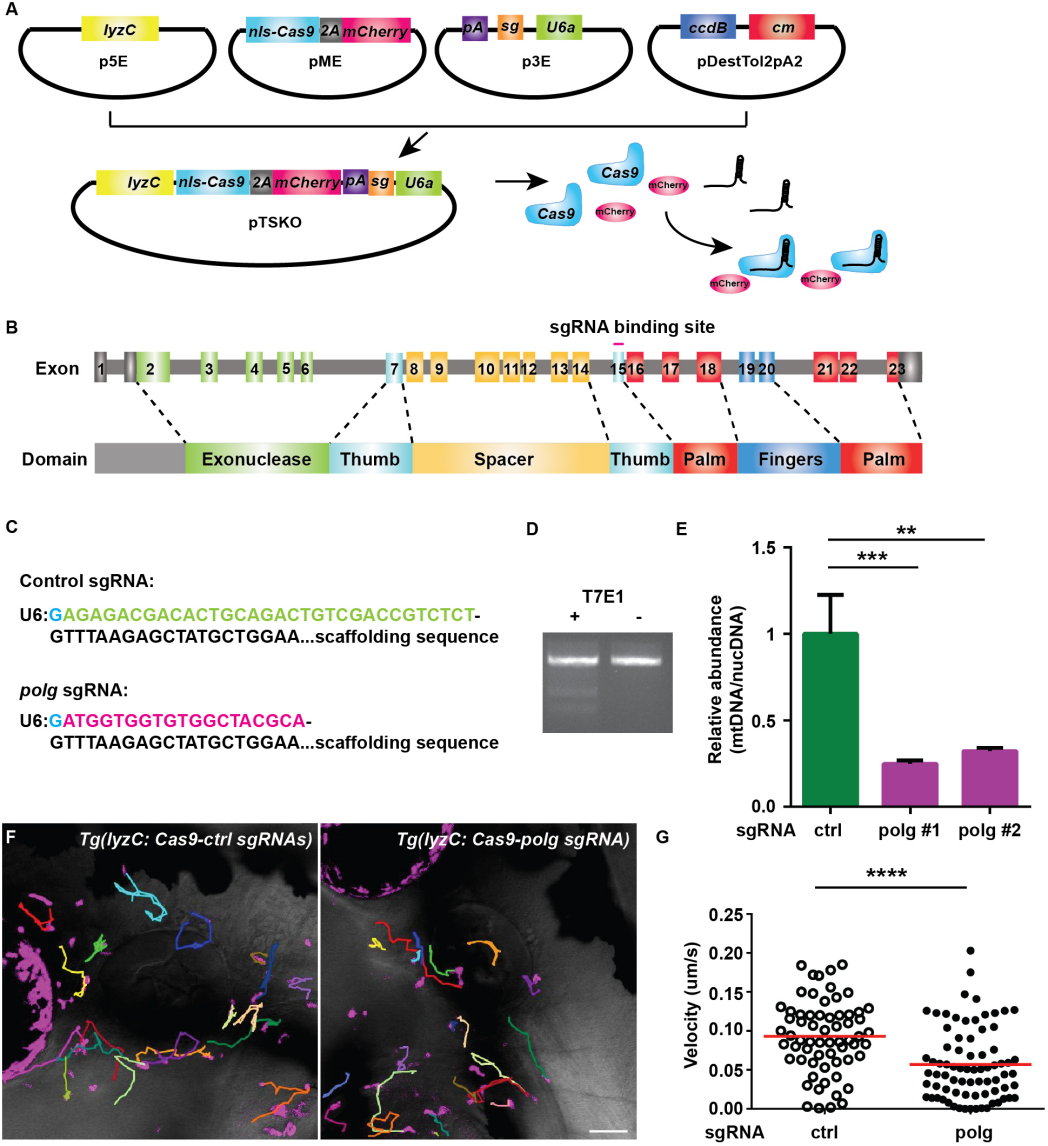
**Neutrophil-specific knockout of *polg* reduced neutrophil motility.** (A). Schematic of the design of gateway vectors to clone constructs for neutrophil-specific knockout. (B) Schematic of the gene structure and protein domains of zebrafish *polg* gene. The sgRNA targets exon 15 in the forward strand. (C) Sequences of sgRNAs (control or *polg*) produced using our vectors. Note that the first G (blue) is included in the backbone. (D,E) Neutrophils were sorted from 3 dpf embryos of *Tg(lyzC:nls-cas9-2A-mCherry/U6a/c:control sgRNA)^pu15^* (TS-ctrl) and *Tg(lyzC:nls-cas9-2A-mCherry/U6a:polg sgRNA)^pu16^* (TS-polg) lines*.* T7E1 assay was performed to show the *in vivo* editing of the *polg* locus (D), and the ratio of mitochondrial DNA/nuclear DNA content was measured by qPCR in two separate sorts (E). ***P<0.001, **P<0.01 by one-way ANOVA. (F,G) Representative images (F) and quantification (G) of neutrophil motility in the head mesenchyme of 3 dpf larvae. One representative result from three biological repeats is shown. *n*=64 for TS-ctrl (ctrl) and *n*=76 for TS-polg (polg) from four different larvae. ****P<0.0001 by Mann–Whitney test. Scale bar: 50 µm.

To disrupt mitochondrial function genetically, elements in the CRISPR/Cas9 vector developed by the Zon group (Ablain et al., 2015) were separately cloned into gateway entry vectors, to facilitate promoter switching and single-guide RNA (sgRNA) cloning. Briefly, the p5E vector contains a tissue-specific promoter to restrict the expression of the Cas9 protein. The pME vector contains DNA encoding a Cas9 protein with nuclear localization signals, linked with mCherry via a self-cleaved 2A peptide to label the target cells. The p3E vector contains U6 promoters and a common scaffold to allow transcription of gene-specific sgRNAs. An SV40 polyA sequence to stabilize the *cas9-2A-mCherry* transcript is also in the p3E. Because zebrafish have had a genome duplication event, we designed the p3E to harbor one or two U6-scaffold cassettes to allow simultaneous expression of two sgRNAs for knocking out both paralogs when needed. Two different U6 promoters, U6a and U6c (Yin et al., 2015), were used to reduce the frequency of homologous recombination of repeat sequences. After gateway recombination, a single plasmid is obtained that allows expression of mCherry and Cas9 in the selected tissue and ubiquitous expression of two sgRNAs for tissue-specific knockouts (Fig. 2A). For a control, a longer sequence without predicted binding sites in the zebrafish genome was used, based on the observation that longer sgRNAs have greatly reduced efficiency (Doench et al., 2014) (Fig. 2C).

### Mitochondrial DNA polymerase regulates neutrophil motility *in vivo*

The first mitochondria-related gene selected for *in vivo* characterization was *polg*, a nuclear-encoded gene that encodes the catalytic subunit of the DNA polymerase gamma, the only DNA polymerase for mitochondrial DNA synthesis. Pathogenic variants of *POLG* cause *POLG*-related disorders, presenting a broad clinical spectrum in human patients (Stumpf et al., 2013). To determine whether *polg* regulates neutrophil motility, a sgRNA against *polg* was selected using CRIPSRScan for neutrophil-specific inhibition (Moreno-Mateos et al., 2015). It targets exon 15 that encodes the Thumb, the catalytic domain, with no predicted off-target sites (Fig. 2B,C). A control line, *Tg(lyzC:nls-cas9-2A-mCherry/U6a/c:control sgRNA)^pu15^* (TS-ctrl), and the transgenic line targeting *polg* in neutrophils, *Tg(lyzC:nls-cas9-2A-mCherry/U6a:polg sgRNA)^pu16^* (TS-polg), were generated. We obtained two founders for each line and the fish were raised to F2 generation. Both lines have similar numbers of neutrophils at 3 dpf. To confirm that *polg* was edited in neutrophils, we isolated the neutrophils from the stable lines and confirmed *in vivo* editing in the neutrophil DNA from the TS-polg line using the T7E1 mutagenesis detection assay (Fig. 2D). We further deep sequenced this locus and detected an average mutation efficiency of 39.52% in two individually sorted samples with in-frame (46.57%) and out-of-frame (53.43%) mutations resulting from deletions or insertions (Fig. S1), likely to have resulted from nonhomologous end joining when DNA double-strand breaks caused by CRISPR/Cas9 were repaired (Lindsay et al., 2016). In addition, the relative abundance of the mitochondrial DNA was significantly reduced in neutrophils sorted from the TS-polg line compared with those sorted from the TS-ctrl line (Fig. 2E). We then quantified the velocity of neutrophil motility in the head mesenchyme. A significant reduction in speed was detected in the TS-polg line at both 3 dpf and 5 dpf (Fig. 2F,G; Fig. S2, Movie 5), indicating that *polg* is required for optimal cell migration.

### The mitochondrial electron transport chain regulates neutrophil motility *in vivo*

**Fig. 3.**
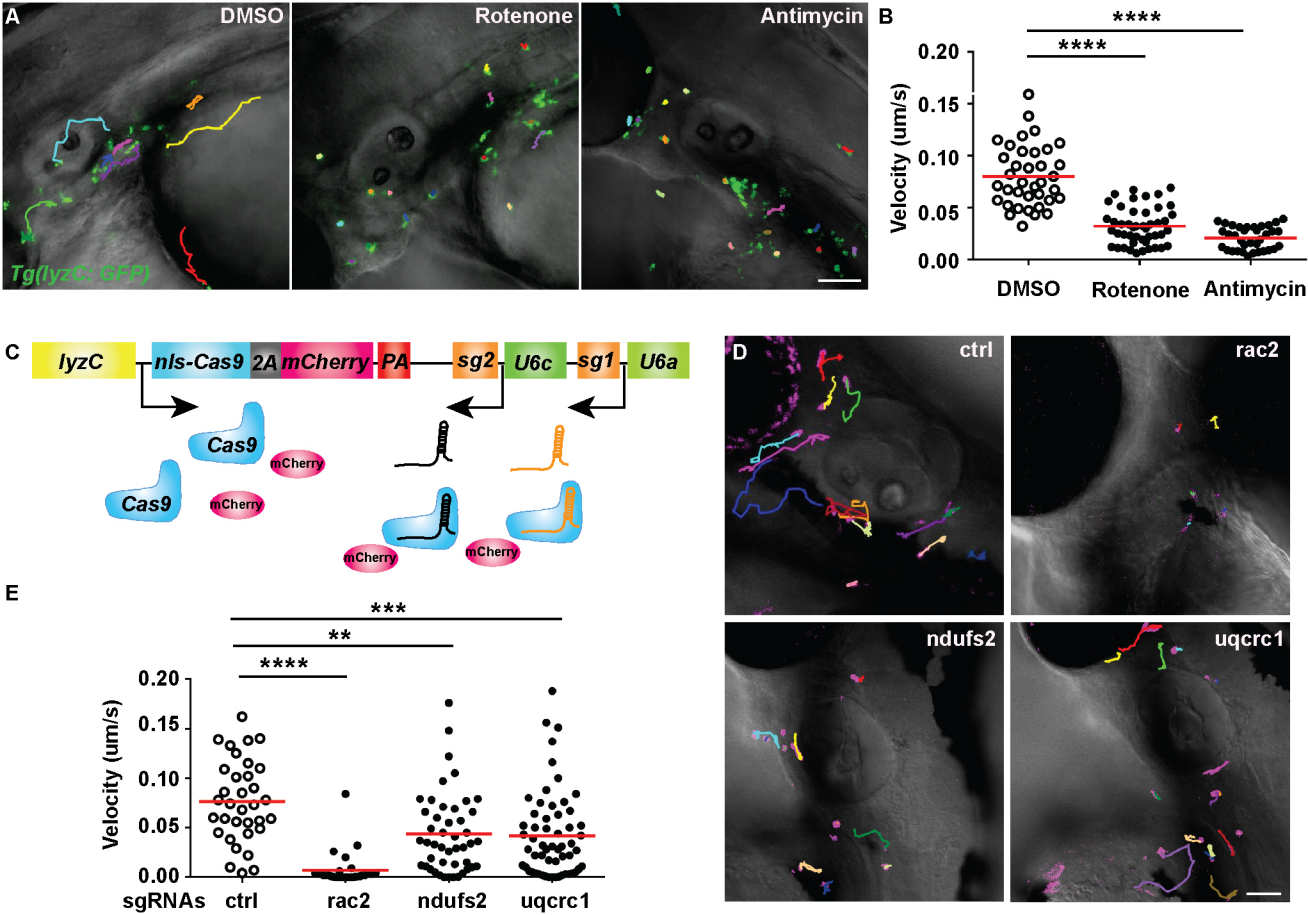
**Mitochondria complex I and III regulate neutrophil motility.** (A,B) *Tg(lyzC:GFP)* at 3 dpf was treated with DMSO, rotenone or antimycin. Representative images (A) and quantification (B) of neutrophil motility in the head mesenchyme. One representative result from three biological repeats is shown. *n*=37 for DMSO, *n*=46 for rotenone and *n*=35 for antimycin from four different larvae. ****P<0.0001 by Kruskal–Wallis test. (C) Schematic of the vector used to produce two different sgRNAs. (D,E) Representative images (D) and quantification (E) of neutrophil motility in the head mesenchyme with neutrophil-specific transient knockout of *rac2* (positive control), *ndufs2* or *uqcrc1*. One representative result from three biological repeats is shown. *n*=34 for control (ctrl), and *n*=30 for *rac2*, *n*=46 for *ndufs2* and *n*=58 for *uqcrc1* knockouts from four different larvae. ****P<0.0001, ***P<0.001, **P<0.01 by Kruskal–Wallis test. Scale bars: 50 µm.

One of the consequences of *polg* loss of function and depletion of mitochondrial DNA is the reduced enzymatic activity of the electron transport chain (ETC) components, 13 of which are encoded by the genes on the mitochondrial DNA (Anderson et al., 1981). The mitochondria uncouplers carbonyl cyanide-4-(trifluoromethoxy)phenylhydrazone (FCCP) and carbonyl cyanide m-chlorophenyl hydrazone (CCCP) rapidly disrupted mitochondria inner membrane potential and abrogated neutrophil chemotaxis in primary human neutrophils *ex vivo* (Bao et al., 2015; Fossati et al., 2003). The complex I inhibitors rotenone and metformin significantly reduced neutrophil recruitment in an acute lipopolysaccharide-induced lung inflammation mouse model (Zmijewski et al., 2008), although this treatment was not neutrophil targeted. We then determined whether proper function of mitochondrial ETC is required for neutrophil motility *in vivo*. FCCP or CCCP treatment resulted in rapid fish death, precluding the suitability of the mitochondria uncouplers for *in vivo* use. Mitochondria complex I inhibitor rotenone, and complex III inhibitor antimycin, significantly reduced neutrophil motility in zebrafish (Fig. 3A,B; Movie 6). Similar to the observations of human primary neutrophils *ex vivo* (Zmijewski et al., 2008), inhibitor treatment did not trigger cell apoptosis.

To further confirm that mitochondrial ETC is essential for neutrophil migration, we disrupted it genetically. Neutrophils migrate individually, allowing functional characterization of a subset of genetically perturbed cells without generating stable lines. To test the feasibility of transient gene disruption *in vivo*, we injected the one-cell stage embryos with a plasmid carrying two different sgRNAs targeting *rac2*, a gene required for neutrophil motility (Deng et al., 2011; Rosowski et al., 2016) (Fig. 3C). At 3 dpf, larvae containing ∼5-10 mCherry-expressing neutrophils in the head mesenchyme were selected for live imaging and quantification. A majority of these neutrophils lost motility (Fig. 3D,E), demonstrating the effectiveness of the tissue-specific knockout strategy *in vivo*. We then designed sgRNAs against *ndufs2* and *uqcrc1*, core components in complex I and III, respectively (Mimaki et al., 2012; Smith et al., 2012). Transient knockout of these two genes also resulted in a reduction in neutrophil motility (Fig. 3D,E; Movie 7), consistent with the results obtained using the pharmacological inhibitors.

### Mitochondrial redox status regulates neutrophil motility *in vivo*

**Fig. 4.**
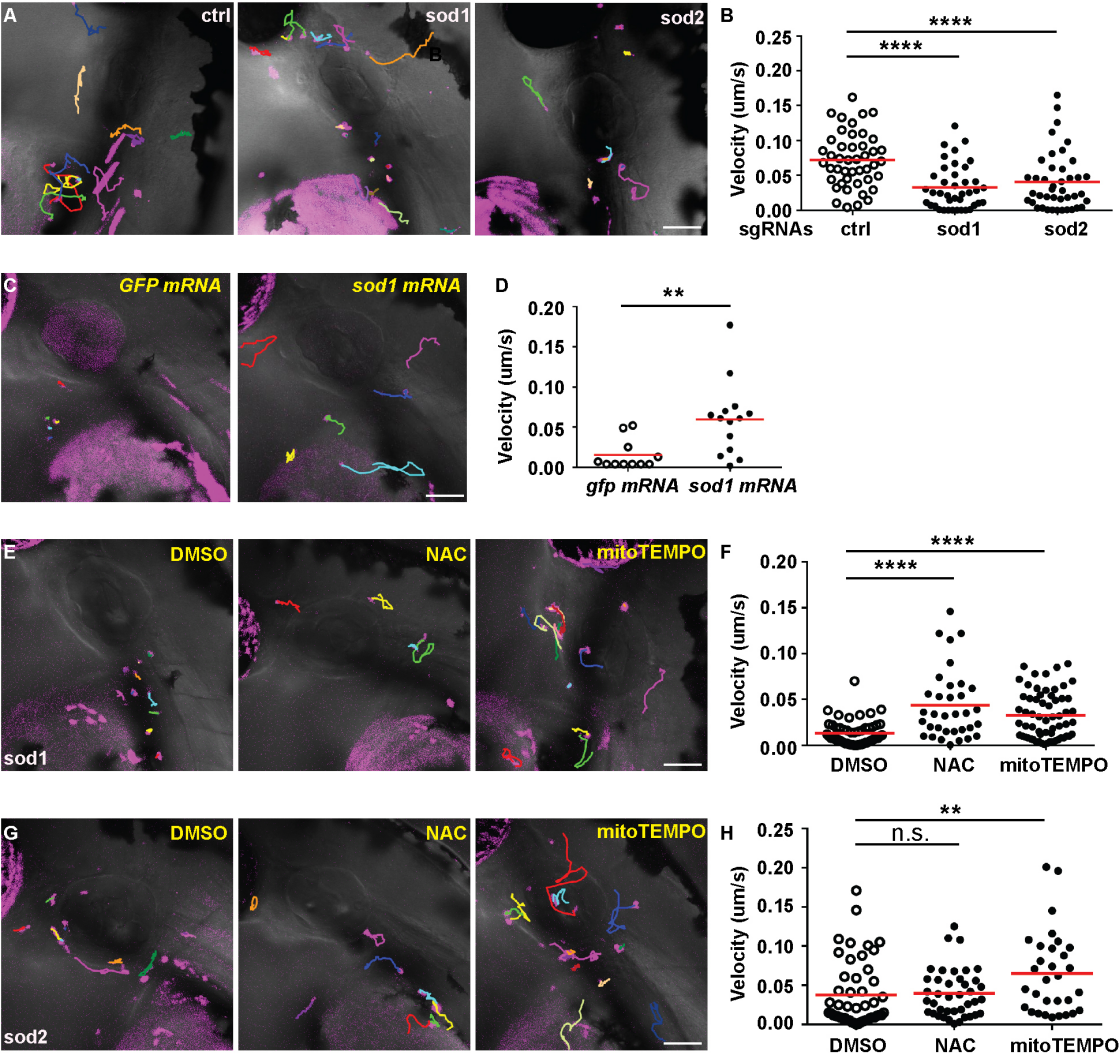
**Mitochondria redox status regulates neutrophil motility.** (A,B) Representative images (A) and quantification (B) of neutrophil motility in the head mesenchyme with neutrophil-specific transient knockout of *sod1* and *sod2* using the plasmids described in Fig. 3C. One representative result from three biological repeats is shown. *n*=36 for control (ctrl), and *n*=40 for *sod1* and *n*=42 for *sod2* knockouts from four different larvae. ****P<0.0001 by Kruskal–Wallis test. (C,D) Representative images (C) and quantification (D) of neutrophil motility in embryos co-injected with the plasmid for *sod1* knockout in neutrophils and *sod1* mRNA or the *GFP* control mRNA. One representative result from three biological repeats is shown. *n*=11 for *gfp* mRNA and *n*=14 for *sod1* mRNA from four different larvae. **P<0.01 by Mann–Whitney test. (E,F) Representative images (E) and quantification (F) of neutrophil motility in embryos injected with the plasmid for *sod1* knockout in neutrophils and then treated with 1% DMSO, 200 µM NAC or 50 µM mitoTEMPO from 1 dpf. One representative result from three biological repeats is shown. *n*=53 for DMSO, *n*=34 for NAC and *n*=62 for mitoTEMPO from four different larvae. ****P<0.0001 by Kruskal–Wallis test. (G,H) Representative images (G) and quantification (H) of neutrophil motility in embryos injected with the plasmid for *sod2* knockout in neutrophils and then treated with 1% DMSO, 200 µM NAC or 50 µM mitoTEMPO from 1 dpf. One representative result from three biological repeats is shown. *n*=49 for DMSO, *n*=40 for NAC and *n*=31 for mitoTEMPO from four different larvae. **P<0.01; n.s., not significant, by Kruskal–Wallis test. Scale bars: 50 µm.

Mitochondria complex I and III directly leak electrons to oxygen molecules and are the major source of intracellular ROS in many cell types. Treating neutrophils *ex vivo* with rotenone increases the intracellular levels of superoxide and hydrogen peroxide (Zmijewski et al., 2008). We then determined whether mitochondrial redox status affects neutrophil motility. Using the same experimental design as in Fig. 3C, we transiently expressed constructs that specifically knock out *sod1* (superoxide dismutase 1 localized in the cytosol and intermembrane space of mitochondria) (Milani et al., 2011) and *sod2* (superoxide dismutase 2 localized in mitochondria matrix) (Peterman et al., 2015) in neutrophils. Both Sod1 and Sod2 mediate the reduction of superoxide (O_2_^−^). Neutrophils with loss of function in *sod1* and *sod2* displayed significantly reduced motility (Fig. 4A,B; Movie 8). The defect in neutrophil motility resulting from *sod1* knockout can be rescued with *sod1* mRNA overexpression (Fig. 4C,D), demonstrating the specificity of the knockout approach. In addition, the reduction of neutrophil motility induced by *sod1/2* deficiency was also rescued by the ROS scavenger N-acetylcysteine (NAC) and/or a mitochondria-targeted antioxidant mitoTEMPO (Fig. 4E-H), indicating that neutrophil motility is inhibited by excessive ROS when *sod1* or *sod2* is knocked out. However, we could not use *sod2* mRNA to rescue the neutrophil-specific disruption of *sod2*, probably because (1) fish embryos cannot tolerate a high level of *sod2* overexpression, or (2) the mutated proteins form aggregates and act as dominant negative (Perry et al., 2010). In contrast, treating zebrafish with pharmacological inhibitors to reduce ROS levels, or overexpression of Sod2 did not affect neutrophil motility (Fig. S3; Movies S9 and 10).

## DISCUSSION

Technically, we have explored the CRISPR/Cas9 system for leukocyte-specific knockout in zebrafish for the first time. We have optimized the original vector developed by the Zon group (Ablain et al., 2015) to allow easier switching of tissue-specific promoters, visualization of Cas9-expressing cells and incorporation of two sgRNAs. The stable TS-polg line generated here displayed an inheritable phenotype, from founders to the F2 generation. The *lyzC* promoter drives Cas9 expression primarily in neutrophils starting at 2 dpf (Hall et al., 2007). At 3 dpf, the editing of the *polg* locus was detected, suggesting the fast-acting nature of this system. However, mutation efficiency estimated using the T7E1 assay or deep sequencing is skewed by the necessary PCR amplification step. Another caveat with this stable line is that the phenotype disappears when crossed with other transgenic lines harboring the *lyzC* or the *mpx* promoter, but not with other lines. We speculate that the presence of another transgene after a neutrophil-specific promoter competes with the transcription of Cas9 and thus reduces the efficiency of target editing. Further optimizations, such as using the GAL4-UAS system, should be attempted in the future. Linking Cas9 with mCherry allows the visualization of the edited cells after transient expression without generating stable lines, significantly increasing the efficiency of scientific discovery. We chose mCherry so the generated tissue-specific knockout line can be used in conjunction with many GFP reporter lines in the zebrafish community. We have demonstrated the efficacy with sgRNAs against *rac2* and two mitochondrial ETC components. We have also demonstrated the specificity by mRNA and chemical rescue of the *sod1* disruption, although not all genes can necessarily be rescued with mRNA expression. We expect that a similar strategy can be used to assess gene functions in other tissues. Caution should be applied when interpreting negative results as it is extremely difficult to evaluate the efficiency of a transient knockout *in vivo*; a conclusion that a gene is not related to the phenotype under investigation should not thus be reached.

Here, we have provided the first evidence that *polg* is required for cell migration. Previously, the characterization of the immune function in *POLG*-related disorders was limited to the adaptive immune system. A heterozygous missense mutation of the *POLG* gene was identified in an infant with decreased T cell number and immunoglobulin numbers, although the significance is not clear (Reichenbach et al., 2006). Increased peripheral lymphocyte death is observed in patients with *POLG*-related disorders (Formichi et al., 2016), suggesting a role for *POLG* in maintaining the pool of adaptive immune cells. Unlike the adaptive immune cells, neutrophils are terminally differentiated and short lived. It is likely that POLG performs functions in neutrophils other than promoting long-term survival. It has been recently noted that POLG localizes to ER-mitochondria contact sites, coinciding with mitochondria fission (Lewis et al., 2016), providing another possible mechanism for POLG in regulating cell migration.

The mitochondrial network we observed in neutrophils *in vivo* is significantly different from those observed in 2D. In isolated primary human neutrophils, mitochondria are devoid from the uropod, and a small fraction with higher membrane potential localizes to cell protrusions (Bao et al., 2015). In contrast, mitochondria localize to the uropod of T cells and neutrophil-like cells (Campello et al., 2006). Mitochondria are recruited to the cell front in highly metastatic breast cancer cells to promote lamellipodia formation, migration and invasion (Zhao et al., 2013). It is possible that both the formation of cell protrusion and tail contraction *in vivo* require proper mitochondria function, whereas mitochondria do not necessarily enter the structures. This tubular structure is consistent with that previously reported in primary human neutrophils (Maianski et al., 2002) and in HL-60 cells (Maianski et al., 2004). In T cells and cancer cells, mitochondria fragmentation promotes cell motility, presumably by increasing proper mitochondria localization and local ATP concentration (Campello et al., 2006; Zhao et al., 2013). It is not clear whether this highly fused network benefits neutrophil migration. A fused network reduces mitophagy (Gomes et al., 2011) and promotes efficient ATP production (Rambold et al., 2015), which might be required to maintain cell homeostasis in addition to regulating cell migration.

Here, we have shown that excessive ROS is detrimental to neutrophil migration, possibly as a result of oxidation of the cytoskeleton or signaling molecules. Despite multiple attempts using several ROS dyes, such as CM-H2DCFDA and CellROX Green, and the genetically encoded ROS sensor Hyper, we failed to label intraneutrophil ROS to show that indeed the ROS levels are increased in *sod1/2* knockouts. Because the biochemical functions of *sod1* and *sod2* are well characterized and the phenotypes are rescued with ROS scavenger treatment (Fig. 4), we are confident that the motility defect has resulted from increased ROS levels. In contrast, increases in ROS production, resulting from mutations that lead to mitochondrial dysfunction (Frezza et al., 2011), pharmacological mitochondria electron transport inhibition with rotenone, or metabolic switch (Porporato et al., 2014), promotes cancer cell migration and invasion. Taken together, the physiological relevance of mitochondrial ROS levels and the dynamics of mitochondria networks are highly context dependent, displaying different features in various cell types in 2D and 3D, warranting further characterization in neutrophils.

In summary, we have characterized the localization of the mitochondria network in zebrafish neutrophils and provided the first evidence of the biological relevance of mitochondria in neutrophil migration in 3D. In addition, we have provided an animal model that allows rapid assessment of the contribution of developmental essential genes in neutrophil migration *in vivo*. We expect that a full understanding of the different contributions of mitochondria-related genes in neutrophil migration will provide mechanistic insights into immune deficiencies seen, and often overlooked, in patients with mitochondrial diseases.

## MATERIALS AND METHODS

### Animals

The zebrafish experiment was conducted in accordance with internationally accepted standards. The Animal Care and Use Protocol was approved by the Purdue Animal Care and Use Committee (PACUC), adhering to the Guidelines for Use of Zebrafish in the National Institutes of Health (NIH) Intramural Research Program (protocol number 1401001018). To generate transgenic zebrafish lines, plasmids with the Tol2 backbone were coinjected with Tol2 transposase mRNA into embryos of the AB strain at one-cell stage as described (Deng et al., 2011).

### Plasmids

All constructs were generated by gateway cloning using LR Clonase II Plus enzyme (Invitrogen). The p5E-lyzC entry vector was generated by replacing the ubiquitin promoter with the *lyzC* promoter in pENTR5′_ubi (Addgene #27320). pENTR5′_ubi was digested by XhoI and BamHI, and the *lyzC* promoter was amplified with primers p5E-lyzC-F/R. In-Fusion cloning (In-Fusion HD Cloning Plus Kit, Clontech) was used to fuse the *lyzC* fragment with the linearized backbone. To make pME-Cas9-2A-mCherry, Cas9 was amplified using pME-Cas9-T2A-GFP (Addgene #63155) as the template with primers pME-Cas9-F/R. Primers 2A-mCherry-F/R were used to amplify 2A-mCherry. Both Cas9 and 2A-mCherry were fused into the pME backbone linearized by SalI and BamHI with In-Fusion cloning. For the p3E-U6a plasmid, U6a was amplified by primers p3E-U6a-F/R using pU6a:sgRNA#2 (Addgene #64246) as the template and inserted into a p3E backbone with polyA. Similarly, both U6a and U6c [using pU6c:sgRNA#4 (Addgene #64248) as the template] were amplified and fused into the p3E backbone with polyA to generate p3E-U6a-U6c using primers p3E-U6ac-F/R and p3E-U6c-F/R, respectively.

A detailed protocol for cloning one or two sgRNAs into the backbones is included in the Supplementary Materials and Methods. *rac2* (ENSDARG00000038010), *ndufs2* (ENSDARG00000007526), *uqcrc1* (ENSDARG00000052304), *sod1* (ENSDARG00000043848) or *sod2* (ENSDARG00000042644) were cloned into p3E-U6a-U6c. The final constructs were assembled using the gateway reactions with the destination vector (pDestTol2pA2) from the Tol2Kit (Kwan et al., 2007).

To generate p5E-DsRed-biUAS, DsRed-biUAS was amplified from pT2-pA-DsRed-T4-E1b-UAS-E1b-Htau40-p301L-PA-2 (a gift from Dr Bettina Schmid, German Center for Neurodegenerative Diseases, Germany) using primers DsRed-biUAS-F/R, and inserted into p5E backbone. For the pME-Sod2-2A-Catalase construct, Sod2-2A-Catalase was amplified with primers Sod2-2A-Catalase-F/R using CMV-Sod2-2A-Catalase (Addgene #67635) as the template and fused into pME backbone. The destination vector pDestR4R2pA (Lawson Lab #465) was used to generate the final expression plasmids. The primers are listed in Table S1.

### Microinjection

Microinjections were performed as described (Deng et al., 2011). Briefly, 1 nl of a mixture containing 25 ng/µl plasmid and 35 ng/µl Tol2 transposase mRNA was injected into the cytoplasm of embryos at one-cell stage.

### Live imaging

Time-lapse spinning disk confocal microscopy (SDCM) was performed with a Yokogawa scanning unit (CSU-X1-A1) mounted on an Olympus IX-83 microscope, equipped with a 100×/1.45 NA UPlanSApo oil objective (Olympus) and an Andor iXon Ultra 897BV EMCCD camera (Andor Technology). GFP and mCherry were excited with 488 nm and 561 nm and fluorescence emission collected through 525/30-nm and 607/36-nm filters, respectively, to determine the localization of mitochondria in migrating neutrophils *in vivo*. Images were captured using MetaMorph version 7.8.8.0 software at 10 s intervals for 5 min. Time-lapse fluorescence images for neutrophil motility were obtained by a laser scanning confocal microscope (LSM 710, Zeiss) with a 20× objective at 1-min intervals for 30 min. The velocity of neutrophils was quantified using ImageJ with MTrackJ plugin and plotted in Prism 6.0 (GraphPad).

### mRNA rescue

To make *sod1* mRNA, the cDNA of *sod1* was amplified from zebrafish total RNA with the following primers using a SuperScript III one-step RT-PCR system with a Platinum *Taq* High Fidelity Kit (Invitrogen): pcs2-sod1-F: 5′-TCTTTTTGCAGGATCCTCGCCACCATGGTGAACAAGGCCGTTT-3′, pcs2-sod1-R: 5′-GTTCTAGAGGCTCGAGTCACTGAGTGATGCCGATC-3′. The purified PCR products were then inserted into the PCS2 backbone using In-Fusion cloning. To generate mRNA, the final pcs2-sod1 construct was linearized by NotI and used for *in vitro* transcription with a mMESSAGE mMACHINE Sp6 kit (Life Technologies). Then, 1 nl of mixture containing 25 ng/µl *sod1* knocking out construct, 35 ng/µl Tol2 transposase mRNA and 300 ng/µl *sod1* mRNA was injected into the cytoplasm of embryos at one-cell stage. *GFP* mRNA was used as the control. Neutrophil motility was measured at 3 dpf using confocal microscopy.

### Chemicals

Embryos at 3 dpf were treated with 1% DMSO, 0.1 µM rotenone (Sigma-Aldrich), 0.1 µM antimycin (Sigma-Aldrich), 200 µM diphenyleneiodonium chloride (DPI, Sigma-Aldrich), 10 µM celastrol (Sigma-Aldrich), or 200 µM N-acetylcysteine (NAC, Sigma-Aldrich) for 30-60 min, followed by time-lapse fluorescence imaging for neutrophil motility with a confocal microscope. For chemical rescue, embryos injected with *sod1* or *sod2* knockout construct were treated with 200 µM NAC or 50 µM mitoTEMPO (Cayman Chemical) at 1 dpf for 48 h. Neutrophil motility was measured at 3 dpf using a confocal microscope.

### Wound assay

Embryos at 3 dpf were treated with chemicals for 1 h before tail transection. They were further incubated in the indicated chemicals for 1 h and then fixed with 4% paraformaldehyde. Neutrophils were stained with Sudan Black and the ones adjacent to the wound edge were quantified.

### Fluorescence-activated cell sorting

*Tg(lyzC:nls-cas9-2A-mCherry/U6a/c:control sgRNA)^pu15^* or *Tg(lyzC:nls-cas9-2A-mCherry/U6a:polg sgRNA)^pu16^* embryos at 3 dpf were digested with trypsin to prepare cell suspensions. mCherry-positive cells were sorted by fluorescence-activated cell sorting (FACS) as described (Deng et al., 2011).

### Isolation of DNA and quantitative PCR

Genomic DNA was purified using a QIAamp DNA Mini Kit (Qiagen) from sorted cells, and 10 µg of poly(dA: dT) (InvivoGen) was used as the carrier DNA. Quantitative PCR was performed using FastStart Essential DNA Green Master (Roche) to determine the relative abundance of mitochondrial DNA. Results were normalized to nucleic DNA. Primers for zebrafish mtDNA (forward: 5′-CAAACACAAGCCTCGCCTGTTTAC-3′; reverse: 5′-CACTGACTTGATGGGGGAGACAGT-3′) and nucDNA (forward: 5′-ATGGGCTGGGCGATAAAATTGG-3′; reverse: 5′-ACATGTGCATGTCGCTCCCAAA-3′) were used (Rooney et al., 2015). The efficiencies of the primers were calculated using Real-time PCR Miner and corrected for the relative abundance (Zhao and Fernald, 2005).

### T7 endonuclease I assay

T7 endonuclease I (NEB) was used to detect mutations caused by CRISPR/Cas9. Genomic DNA containing sgRNA recognition site was amplified by PCR from sorted cells. PCR products were purified with a PCR purification kit (Clontech) and reannealed in a thermocycler using the following conditions: 95°C for 5 min, 95-85°C with ramp rate as –0.3°C/s; 85-25°C with ramp rate as –0.1°C/s. Reannealed PCR products were incubated with T7 endonuclease I at 37°C for 1 h, followed by agarose gel electrophoresis. Primers used for this assay were Polg-F/R as described below.

### Mutational efficiency quantification

To determine the mutation efficiency in *Tg(lyzC:nls-cas9-2A-mCherry/U6a:polg sgRNA)^pu16^*, the *polg* locus around the sgRNA binding site was amplified by PCR using primers (Polg-F: 5′-CAGCCAACGTATGCGGCTAC-3′, Polg-R: 5′-CAGCTCTGCGGGTGACTGTTC-3′), followed by library construction using a Nextera Library Prep Kit and sequencing using an Illumina MiSeq 300 at the sequencing center of Purdue University. Raw reads have been deposited to the Sequence Read Archive (accession number SRP132222). Mutational efficiency was calculated using the CrispRVariants R package (Lindsay et al., 2016), with the specific code below to compare aligned reads and count read frequencies:

crispr_set <- readsToTarget(c(“./crisprVariants/2018-01-05_Data/001664pr.bam”, “./crisprVariants/2018-01-05_Data/001675pr.bam”), target=sgRNA40_target, reference=sgRNA40_RevComp_Reference, names=c(“polg #1”,“polg #2”), target.loc=sgRNA40.revC.loc, orientation=“target”, chimera.to.target=25)

The mutational efficiency was calculated using the following command built into CrispRVariants:

mutationEfficiency(crispr_set)

Mutation types were calculated by using the following command built into CrispRvariants that provides consensus sequences of the different mutations found in the two deep sequencing paired-end samples:

consensusSeqs(crispr_set)

The consensus sequences are labeled using the CIGAR string format, and mutational frequencies were determined from that labeling.

### Statistical analysis

Statistical analysis was performed with Prism 6 (GraphPad). An unpaired two-tailed Student's *t-*test or one-way ANOVA was used to determine the statistical significance of differences between groups. A *P*-value less than 0.05 was considered as statistically significant.

## Supplementary Material

Supplementary information

First Person interview

## References

[DMM033027C1] Ablain J., Durand E. M., Yang S., Zhou Y. and Zon L. I. (2015). A CRISPR/Cas9 vector system for tissue-specific gene disruption in zebrafish. *Dev. Cell* 32, 756-764. 10.1016/j.devcel.2015.01.032 25752963 PMC4379706

[DMM033027C2] Anderson S., Bankier A. T., Barrell B. G., de Bruijn M. H., Coulson A. R., Drouin J., Eperon I. C., Nierlich D. P., Roe B. A., Sanger F. et al. (1981). Sequence and organization of the human mitochondrial genome. *Nature* 290, 457-465. 10.1038/290457a0 7219534

[DMM033027C3] Bao Y., Ledderose C., Graf A. F., Brix B., Birsak T., Lee A., Zhang J. and Junger W. G. (2015). mTOR and differential activation of mitochondria orchestrate neutrophil chemotaxis. *J. Cell Biol.* 210, 1153-1164. 10.1083/jcb.201503066 26416965 PMC4586745

[DMM033027C4] Campello S., Lacalle R. A., Bettella M., Mañes S., Scorrano L. and Viola A. (2006). Orchestration of lymphocyte chemotaxis by mitochondrial dynamics. *J. Exp. Med.* 203, 2879-2886. 10.1084/jem.20061877 17145957 PMC2118173

[DMM033027C5] Deng Q. and Huttenlocher A. (2012). Leukocyte migration from a fish eye's view. *J. Cell Sci.* 125, 3949-3956. 10.1242/jcs.093633 23104739 PMC3482313

[DMM033027C6] Deng Q., Yoo S. K., Cavnar P. J., Green J. M. and Huttenlocher A. (2011). Dual roles for Rac2 in neutrophil motility and active retention in zebrafish hematopoietic tissue. *Dev. Cell* 21, 735-745. 10.1016/j.devcel.2011.07.013 22014524 PMC3199325

[DMM033027C7] Desai S. P., Bhatia S. N., Toner M. and Irimia D. (2013). Mitochondrial localization and the persistent migration of epithelial cancer cells. *Biophys. J.* 104, 2077-2088. 10.1016/j.bpj.2013.03.025 23663851 PMC3647149

[DMM033027C8] Doench J. G., Hartenian E., Graham D. B., Tothova Z., Hegde M., Smith I., Sullender M., Ebert B. L., Xavier R. J. and Root D. E. et al. (2014). Rational design of highly active sgRNAs for CRISPR-Cas9-mediated gene inactivation. *Nat. Biotechnol.* 32, 1262-1267. 10.1038/nbt.3026 25184501 PMC4262738

[DMM033027C9] Formichi P., Radi E., Branca C., Battisti C., Brunetti J., Da Pozzo P., Giannini F., Dotti M. T., Bracci L., Federico A. et al. (2016). Oxidative stress-induced apoptosis in peripheral blood lymphocytes from patients with POLG-related disorders. *J. Neurol. Sci.* 368, 359-368. 10.1016/j.jns.2016.07.047 27538665

[DMM033027C10] Fossati G., Moulding D. A., Spiller D. G., Moots R. J., White M. R. H. and Edwards S. W. (2003). The mitochondrial network of human neutrophils: role in chemotaxis, phagocytosis, respiratory burst activation, and commitment to apoptosis. *J. Immunol.* 170, 1964-1972. 10.4049/jimmunol.170.4.1964 12574365

[DMM033027C11] Frezza C., Pollard P. J. and Gottlieb E. (2011). Inborn and acquired metabolic defects in cancer. *J. Mol. Med.* 89, 213-220. 10.1007/s00109-011-0728-4 21301796 PMC3043233

[DMM033027C12] Gomes L. C., Di Benedetto G. and Scorrano L. (2011). During autophagy mitochondria elongate, are spared from degradation and sustain cell viability. *Nat. Cell Biol.* 13, 589-598. 10.1038/ncb2220 21478857 PMC3088644

[DMM033027C13] Hall C., Flores M. V., Storm T., Crosier K. and Crosier P. (2007). The zebrafish lysozyme C promoter drives myeloid-specific expression in transgenic fish. *BMC Dev. Biol.* 7, 42 10.1186/1471-213X-7-42 17477879 PMC1877083

[DMM033027C14] Heggeness M. H., Simon M. and Singer S. J. (1978). Association of mitochondria with microtubules in cultured-cells. *Proc. Natl. Acad. Sci. USA* 75, 3863-3866. 10.1073/pnas.75.8.3863 80800 PMC392888

[DMM033027C15] Kwan K. M., Fujimoto E., Grabher C., Mangum B. D., Hardy M. E., Campbell D. S., Parant J. M., Yost H. J., Kanki J. P. and Chien C.-B. (2007). The Tol2kit: a multisite gateway-based construction kit for Tol2 transposon transgenesis constructs. *Dev. Dyn* 236, 3088-3099. 10.1002/dvdy.21343 17937395

[DMM033027C16] Lam P.-Y., Fischer R. S., Shin W. D., Waterman C. M. and Huttenlocher A. (2014). Spinning disk confocal imaging of neutrophil migration in zebrafish. *Methods Mol. Biol.* 1124, 219-233. 10.1007/978-1-62703-845-4_14 24504955 PMC4087032

[DMM033027C17] Lämmermann T., Bader B. L., Monkley S. J., Worbs T., Wedlich-Söldner R., Hirsch K., Keller M., Förster R., Critchley D. R., Fässler R. et al. (2008). Rapid leukocyte migration by integrin-independent flowing and squeezing. *Nature* 453, 51-55. 10.1038/nature06887 18451854

[DMM033027C18] Lewis S. C., Uchiyama L. F. and Nunnari J. (2016). ER-mitochondria contacts couple mtDNA synthesis with mitochondrial division in human cells. *Science* 353, aaf5549 10.1126/science.aaf5549 27418514 PMC5554545

[DMM033027C40] Lindsay H., Burger A., Biyong B., Felker A., Hess C., Zaugg J., Chiavacci E., Anders C., Jinek M., Mosimann C. and Robinson M. D. (2016). CrispRVariants charts the mutation spectrum of genome engineering experiments. *Nature Biotechnol.* 34, 701-702. 10.1038/nbt.3628 27404876

[DMM033027C19] Maianski N. A., Mul F. P., van Buul J. D., Roos D. and Kuijpers T. W. (2002). Granulocyte colony-stimulating factor inhibits the mitochondria-dependent activation of caspase-3 in neutrophils. *Blood* 99, 672-679. 10.1182/blood.V99.2.672 11781253

[DMM033027C20] Maianski N. A., Geissler J., Srinivasula S. M., Alnemri E. S., Roos D. and Kuijpers T. W. (2004). Functional characterization of mitochondria in neutrophils: a role restricted to apoptosis. *Cell Death Differ.* 11, 143-153. 10.1038/sj.cdd.4401320 14576767

[DMM033027C21] Milani P., Gagliardi S., Cova E. and Cereda C. (2011). SOD1 transcriptional and posttranscriptional regulation and its potential implications in ALS. *Neurol. Res. Int.* 2011, 458427 10.1155/2011/458427 21603028 PMC3096450

[DMM033027C22] Mimaki M., Wang X., McKenzie M., Thorburn D. R. and Ryan M. T. (2012). Understanding mitochondrial complex I assembly in health and disease. *Biochim. Biophys. Acta* 1817, 851-862. 10.1016/j.bbabio.2011.08.010 21924235

[DMM033027C23] Moreno-Mateos M. A., Vejnar C. E., Beaudoin J. D., Fernandez J. P., Mis E. K., Khokha M. K. and Giraldez A. J. (2015). CRISPRscan: designing highly efficient sgRNAs for CRISPR-Cas9 targeting in vivo. *Nat. Methods* 12, 982-988. 10.1038/nmeth.3543 26322839 PMC4589495

[DMM033027C24] Perry J. J., Shin D. S., Getzoff E. D. and Tainer J. A. (2010). The structural biochemistry of the superoxide dismutases. *Biochim. Biophys. Acta* 1804, 245-262. 10.1016/j.bbapap.2009.11.004 19914407 PMC3098211

[DMM033027C25] Peterman E. M., Sullivan C., Goody M. F., Rodriguez-Nunez I., Yoder J. A. and Kim C. H. (2015). Neutralization of mitochondrial superoxide by superoxide dismutase 2 promotes bacterial clearance and regulates phagocyte numbers in zebrafish. *Infect. Immun.* 83, 430-440. 10.1128/IAI.02245-14 25385799 PMC4288898

[DMM033027C26] Porporato P. E., Payen V. L., Pérez-Escuredo J., De Saedeleer C. J., Danhier P., Copetti T., Dhup S., Tardy M., Vazeille T., Bouzin C. et al. (2014). A mitochondrial switch promotes tumor metastasis. *Cell Rep.* 8, 754-766. 10.1016/j.celrep.2014.06.043 25066121

[DMM033027C27] Rambold A. S., Cohen S. and Lippincott-Schwartz J. (2015). Fatty acid trafficking in starved cells: regulation by lipid droplet lipolysis, autophagy, and mitochondrial fusion dynamics. *Dev. Cell* 32, 678-692. 10.1016/j.devcel.2015.01.029 25752962 PMC4375018

[DMM033027C28] Reichenbach J., Schubert R., Horvàth R., Petersen J., Fütterer N., Malle E., Stumpf A., Gebhardt B. R., Koehl U., Schraven B. et al. (2006). Fatal neonatal-onset mitochondrial respiratory chain disease with T cell immunodeficiency. *Pediatr. Res.* 60, 321-326. 10.1203/01.pdr.0000233252.60457.cf 16857757

[DMM033027C41] Rooney J. P., Ryde I. T., Sanders L. H., Howlett E. H., Colton M. D., Germ K. E., Mayer G. D., Greenamyre J. T. and Meyer J. N. (2015) PCR Based Determination of Mitochondrial DNA Copy Number in Multiple Species. *Methods Mol. Biol.* 1241, 23-38. 10.1007/978-1-4939-1875-1_3 25308485 PMC4312664

[DMM033027C29] Rosowski E. E., Deng Q., Keller N. P. and Huttenlocher A. (2016). Rac2 functions in both neutrophils and macrophages to mediate motility and host defense in larval zebrafish. *J. Immunol.* 197, 4780-4790. 10.4049/jimmunol.1600928 27837107 PMC5367389

[DMM033027C30] Smith P. M., Fox J. L. and Winge D. R. (2012). Biogenesis of the cytochrome bc(1) complex and role of assembly factors. *Biochim. Biophys. Acta* 1817, 276-286. 10.1016/j.bbabio.2011.11.009 22138626 PMC3366459

[DMM033027C31] Stumpf J. D., Saneto R. P. and Copeland W. C. (2013). Clinical and molecular features of POLG-related mitochondrial disease. *Cold Spring Harbor Perspect. Biol.* 5, a011395 10.1101/cshperspect.a011395 PMC3683902 23545419

[DMM033027C32] Walker M. A., Slate N., Alejos A., Volpi S., Iyengar R. S., Sweetser D., Sims K. B. and Walter J. E. (2014). Predisposition to infection and SIRS in mitochondrial disorders: 8 years’ experience in an academic center. *J. Aller Cl Imm-Pract.* 2, U465-U173. 10.1016/j.jaip.2014.02.009 25017538

[DMM033027C33] Warburg O. (1956). On respiratory impairment in cancer cells. *Science* 124, 269-270. 13351639

[DMM033027C34] Yamahashi Y., Cavnar P. J., Hind L. E., Berthier E., Bennin D. A., Beebe D. and Huttenlocher A. (2015). Integrin associated proteins differentially regulate neutrophil polarity and directed migration in 2D and 3D. *Biomed. Microdevices* 17, 100 10.1007/s10544-015-9998-x 26354879 PMC4678772

[DMM033027C35] Yin L. L., Maddison L. A., Li M. Y., Kara N., LaFave M. C., Varshney G. K., Burgess S. M., Patton J. G. and Chen W. B. (2015). Multiplex conditional mutagenesis using transgenic expression of Cas9 and sgRNAs. *Genetics* 200, 431-441. 10.1534/genetics.115.176917 25855067 PMC4492370

[DMM033027C36] Zhao S. and Fernald R. D. (2005). Comprehensive algorithm for quantitative real-time polymerase chain reaction. *J. Comput. Biol* 12, 1047-1064. 10.1089/cmb.2005.12.1047 16241897 PMC2716216

[DMM033027C37] Zhao J., Zhang J., Yu M., Xie Y., Huang Y., Wolff D. W., Abel P. W. and Tu Y. (2013). Mitochondrial dynamics regulates migration and invasion of breast cancer cells. *Oncogene* 32, 4814-4824. 10.1038/onc.2012.494 23128392 PMC3911914

[DMM033027C38] Zmijewski J. W., Lorne E., Zhao X., Tsuruta Y., Sha Y. G., Liu G., Siegal G. P. and Abraham E. (2008). Mitochondrial respiratory complex I regulates neutrophil activation and severity of lung injury. *Am. J. Respir. Crit. Care. Med.* 178, 168-179. 10.1164/rccm.200710-1602OC 18436790 PMC2453511

