# Supplementary material for: Neutrophil-specific knockout demonstrates a role for mitochondria in regulating neutrophil motility in zebrafish
Source: Dis Model Mech. 2018 Mar 1;11(3):dmm033027. doi: 10.1242/dmm.033027 (PMC5897731; doi:10.1242/dmm.033027)
Supplement: Supplementary information [file dmm-11-033027-s1.pdf]

FIRST PERSON

First person – Wenqing Zhou

First Person is a series of interviews with the first authors of a selection of papers published in Disease Models & Mechanisms, helping early-career researchers promote themselves alongside their papers. Wenqing Zhou is first author on 'Neutrophil-specific knockout demonstrates a role for mitochondria in regulating neutrophil motility in zebrafish', published in DMM. Wenqing is a graduate student in the lab of Dr Qing Deng at Purdue University, Lafayette, USA, investigating the migration and response of neutrophils to inflammation, using zebrafish as an *in vivo* model.

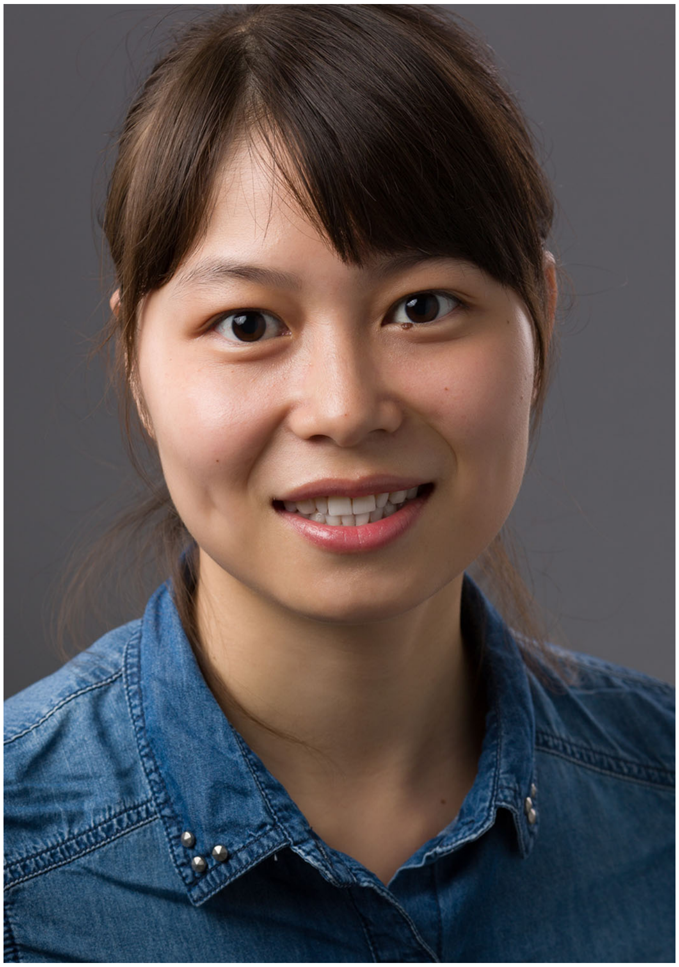

Wenqing Zhou

How would you explain the main findings of your paper to non-scientific family and friends?

Mitochondria, the powerhouses of cells, provide energy for cellular processes. The causes of mitochondrial disorders vary, and can stem from mutations in the genes of mitochondria themselves, or genes in the nucleus that are important for mitochondrial function. Patients with mitochondrial disease have a spectrum of defects mostly noted in the neuronal and muscular systems. A subset of patients are also susceptible to infections, indicating immune deficiency. However, the cause of this immune deficiency is not well understood. Neutrophils are fast-moving cells and the immune system's first line of defense against invading pathogens. Neutrophils are different from other cells in that they primarily rely on energy obtained from cellular processes independent of mitochondria. It is not clear whether and how neutrophil function is impaired in patients with mitochondrial disorders. In order to determine the contribution of mitochondria in neutrophils, we disrupted mitochondria-related genes in the neutrophils of zebrafish. We found a range of mitochondria-specific genes that regulate neutrophil motility. We have now provided the first evidence from a living organism that neutrophil dysfunction is a component in at least a group of mitochondrial disease patients.

What are the potential implications of these results for your field of research?

We have provided evidence that five genes related to mitochondrial function are required for neutrophil migration. Our result suggests that patients with these mutations should be protected from infections, especially those normally controlled by neutrophils such as extracellular bacteria and fungi.

Technically, we demonstrate that our tissue-specific knockout approach could be used to quickly screen for gene function in neutrophils. It is expected that scientific discoveries will be accelerated in this terminally differentiated cell type that is resistant to genetic manipulation *in vitro*.

What are the main advantages and drawbacks of the model system you have used as it relates to the disease you are investigating?

The beauty of zebrafish as a model is that the larvae are transparent, allowing the non-invasive observation of cell behaviors *in vivo*. With the zebrafish genome fully sequenced, we now know that the innate immune system and mitochondria-related genes are conserved between humans and zebrafish. The genetic and pharmacological tools are robust. Combined with a large number of embryos, this is an ideal model organism to screen and discover genes that regulate vertebrate-specific biology such as neutrophil function.

> "[Zebrafish are] an ideal model organism to screen and discover genes that regulate vertebrate-specific biology such as neutrophil function"

The drawback is that we still need to eventually validate our findings in human cells or mice models. Difficulties with tissue-specific knockout have been a major technical problem in the zebrafish research field and although we have made some further improvement here, it is still not perfect. For example, it is not easy to measure editing efficiency, especially with transient expression of the current tissue-specific knock-out system. Rescue may also be

Wenqing Zhou's contact details: Department of Biological Sciences, Purdue University, West Lafayette, IN 47907, USA.
E-mail: zhou522@purdue.edu

very difficult at present in order to demonstrate the specificity of a knockout.

What has surprised you the most while conducting your research?

The neutrophil-specific CRISPR/Cas9 in zebrafish was driven by the lysozyme C promoter, which starts to make transcripts at 2 days post-fertilization (dpf). We were able to observe strong phenotypes (reduced neutrophil velocity or mitochondrial DNA) at 3 dpf, even with transient expression of the system, indicating a very high editing efficiency of this tissue-specific CRISPR/Cas9 system in zebrafish.

Describe what you think is the most significant challenge impacting your research at this time and how will this be addressed over the next 10 years?

Work on essential developmental genes requires tissue-specific disruption. The challenge is that we are not confident that all genes can be disrupted efficiently in order to allow us to use our screen to conclude that they are not required for neutrophil function. In addition, we cannot yet disrupt genes encoded by mitochondrial DNA. An economic and efficient way to estimate cell-specific mutation efficiency during the initial screening will be very powerful. Improved CRISPR precision and flexibility with PAM sequences will help as well.

During the course of our research, we also noted that a lot of tools used in cell culture, such as membrane potential dyes and ROS indicator dyes, do not penetrate to label neutrophils in tissue. Neutrophil-specific lines that encode genetic sensors may help report the physiological status of neutrophils upon gene disruption.

What's next for you?

I will continue to work on evaluating other mitochondria-related genes in neutrophil motility and identifying the underlying chemotaxis pathways regulated by these genes. A full understanding of the contribution of each mitochondria-related gene to neutrophil migration will provide mechanistic insights and instruct precision medicine to treat the immune deficiencies seen, and often overlooked, in patients with mitochondrial diseases.

Reference

Zhou, W., Cao, L., Jeffries, J., Zhu, X., Staiger, C. J. and Deng, Q. (2018). Neutrophil-specific knockout demonstrates a role for mitochondria in regulating neutrophil motility in zebrafish. *Dis. Model. Mech.* **11**: dmm033027, doi:10.1242/dmm.033027.

Disease Models & Mechanisms
